# Using Hand-Held Chlorophyll Meters and Canopy Reflectance Sensors for Fertilizer Nitrogen Management in Cereals in Small Farms in Developing Countries

**DOI:** 10.3390/s20041127

**Published:** 2020-02-19

**Authors:** Bijay-Singh, Ali M. Ali

**Affiliations:** 1 Department of Soil Science, Punjab Agricultural University, Ludhiana 141 004, India; 2 Department of Soil Fertility and Microbiology, Desert Research Center, Cairo 11753, Egypt; alimohamed1982@gmail.com

**Keywords:** rice, wheat, maize, SPAD meter, atLeaf, GreenSeeker, crop circle

## Abstract

To produce enough food, smallholder farmers in developing countries apply fertilizer nitrogen (N) to cereals, sometimes even more than the local recommendations. During the last two decades, hand-held chlorophyll meters and canopy reflectance sensors, which can detect the N needs of the crop based on transmission and reflectance properties of leaves through proximal sensing, have been studied as tools for optimizing crop N status in cereals in developing countries. This review aims to describe the outcome of these studies. Chlorophyll meters are used to manage fertilizer N to maintain a threshold leaf chlorophyll content throughout the cropping season. Despite greater reliability of the sufficiency index approach, the fixed threshold chlorophyll content approach has been investigated more for using chlorophyll meters in rice and wheat. GreenSeeker and Crop Circle crop reflectance sensors take into account both N status and biomass of the crop to estimate additional fertilizer N requirement but only a few studies have been carried out in developing countries to develop N management strategies in rice, wheat and maize. Both chlorophyll meters and canopy reflectance sensors can increase fertilizer N use efficiency by reduction of N rates. Dedicated economic analysis of the proximal sensing strategies for managing fertilizer N in cereals in developing countries is not adequately available.

## 1. Introduction

**Figure 1:**
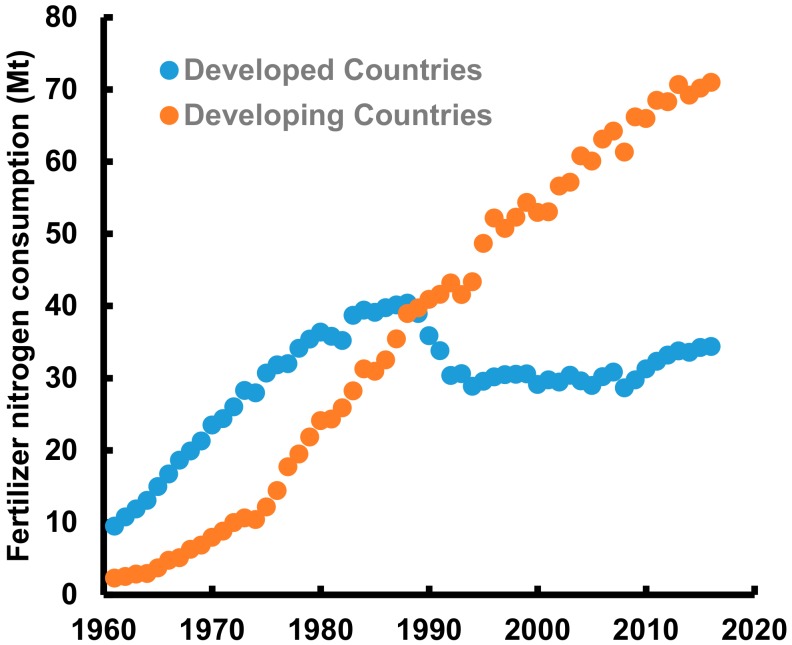
Total fertilizer nitrogen consumption in developed and developing countries since the 1960s. Data source: [2].

During the next half century, most of the developing countries in the world will continue striving for increased food production by using mineral fertilizers [1] and will continue to use fertilizers for food production. Until 1989, total fertilizer nitrogen (N) consumption kept increasing in both developed and developing countries but afterwards while an actual decrease was observed in the developed countries, the increasing trend is still continuing for developing countries (Figure 1). In 2016, 71.0 and 34.4 Mt of fertilizer N was consumed in developing and developed countries, respectively [2]. Low population pressure as well as the adoption of farming practices leading to high fertilizer N use efficiency constitute the two main factors affecting reduced consumption of fertilizer N in the developed world. In 2016, India and China, the two most populous developing countries in the world used 25% and 16%, respectively, of the total fertilizer N consumed globally. Because N fertilizers are heavily subsidized, farmers in developing countries tend to use excessive quantities to ensure high crop yields without any consideration of the low N use efficiency and environmental and soil health cost.

That 74.5% of the global population of 7.72 billion in July 2019 lives in developing countries is one factor behind continuously increasing fertilizer N consumption in developing countries; low fertilizer N use efficiency in smallholder farms in these countries could be another. According to the International Fund for Agricultural Development, a small-scale farm is defined as the one with land area of two hectares or less [3]. According to an estimate, there are about 450 million small-scale farms all over the world supporting a population of roughly 2.2 billion people, mostly in developing countries [4]. In India, respectively 48.7%, 43.4% and 7.4% irrigated land under cultivation consists of small (less than 2 ha), medium (2–10 ha) and large (more than 10 ha) size farms, and average fertilizer N use in the three categories was 148.9, 108.5 and 114.6 kg N ha^−1^, respectively [5]. Ju et al. [6] and Ren et al. [7] reported that fertilizer use per unit area sharply decreased with increasing farm size in China. The yield of crops was higher in big farms than in smallholder farms [6]. Wu et al. [8] observed a 0.3% decrease in fertilizer use with a 1% increase in farm size. Due to the high costs of fixed inputs (such as machinery), smallholders tend to use more non-fixed inputs such as N fertilizers than fixed inputs to maximize cereal production [9], thereby leading to low fertilizer N use efficiency. Smallholder farms occupy up to 40% of agricultural areas globally [10] where rational and need based application of fertilizer N can ensure high crop production levels with minimal risk of fertilizer-related environmental pollution.

In developing regions of the world, fertilizer N is generally managed in the form of a blanket or standard recommendations formulated by agronomists and soil scientists by averaging the crop response data collected over large geographic areas having similar climate and land forms. In standard fertilizer N recommendations, based on the expected crop response to fertilizer N, amount and timing of N applications are prescribed before planting. The standard recommendations cannot take into account the dynamic spatial variability in N supplying capacity of soils during the crop growth. Because standard fertilizer N recommendations are designed to produce optimum yields in *all* the fields in the region, these may lead to excessive N application in several fields. Lower fertilizer N use efficiencies, lower profits and increased risk of environmental degradation due to loss of unutilized N through leaching or volatilization are often linked with application of more fertilizer than needed by the crop [11]. In the quest to achieve high yields, many times uninformed farmers apply fertilizer N even more than the standard recommendations. It leads to further lowering of fertilizer use efficiency.

Fertilizer N use in cereals can be best optimized through increased synchronization between crop N demand and the supply of N from different sources throughout the crop growing season on a field-specific or site-specific basis [12,13]. As an alternative to standard fertilizer recommendations, the site-specific N management approach explicitly recognizes the need to efficiently utilize indigenous N in the soil. It aims to optimally match the requirements of crops by applying adequate amounts of N at appropriate times during the crop growth period. It is now possible to optimize fertilizer N management to account for the variations in the N supplying capacity of the soil through variable rate N-fertilizer application in large fields in developed countries and by determining field-specific fertilizer N application rates in small fields in developing countries [14,15,16,17,18,19,20]. The on-the-go crop sensing/variable-rate fertilizers N applicators based on spatially variable needs for N at critical growth stages of crops as being used in large fields in developed countries are not suitable for farmers in developing regions because a majority of them possess small land holdings.

In developing countries, farmers often use leaf greenness as a subjective indicator of N needs of cereals even though visual estimate of leaf greenness is influenced by sunlight variability and it cannot be precisely quantified. To assess the real time and site-specific N needs of crop plants, quantitative estimation of N status of the leaves using appropriate diagnostic tools constitutes an essential element [21]. Because spectral characteristics of radiation reflected, transmitted or absorbed by intact leaves can provide an estimate of leaf chlorophyll content [22], technological advances have enabled the development of a number of optical sensors that non-invasively measure light transmittance or reflectance to estimate crop N status. Among these sensors, hand-held chlorophyll meters, such as Soil Plant Analysis Development (SPAD) 502 plus (Konika Minolta^®^ Inc., Tokyo, Japan) and atLEAF (FT Green LLC^®^, Wilmington, DE, USA), and hand-held canopy reflectance sensors such as GreenSeeker (Trimble Inc., Sunnyvale, CA, USA) and Crop Circle (Holland Scientific^®^ Inc., Lincoln, NE, USA), are now available for use in developing countries to manage fertilizer N as per need of the crop when it is already in the field. The canopy reflectance sensors measure visible and near-infrared (NIR) spectral response from crop canopies and the output is expressed as vegetation indices such as Normalized Difference Vegetation Index (NDVI). Both chlorophyll meters and canopy reflectance sensors are used either in contact with or within 2 m of the target following principles of proximal sensing [23] and can guide field-specific mid-season fertilizer N application in different field crops [24]. The objective of this review is to describe protocols for using different hand-held chlorophyll meters and canopy reflectance sensors for field specific N management in cereals and review the performance of different devices in terms of improving fertilizer N use efficiency in developing regions of the world. Strengths and limitations of the two categories of the devices in guiding need based N management have also been discussed.

## 2. Development of Proximal Sensing with Chlorophyll Meters and Canopy Reflectance Sensors for Fertilizer N Management in Cereal

Managing fertilizer N in cereals based on proximal sensing of spectral properties of leaves is relatively a recent development. Reflectance properties of canopies or transmittance properties of leaves as measured by proximal sensing offer great advantages of speed and immediacy in fertilizer N management of crops. Hand-held transmission meters, commonly referred to as chlorophyll meters, are used to measure transmittance of different wavelength lights through plant leaves to estimate the relative chlorophyll content of leaves. Numerical chlorophyll meter readings are unit less but being proportional to the amount of chlorophyll present in the leaf, provide a relative rather than an absolute measure of the chlorophyll content. On the other hand, crop reflectance sensors measure vegetation indices from reflectance of visible and NIR radiations from crop canopies. As vegetation indices may be related to chlorophyll content and biomass, these are used to assess crop N status and establish relationships with fertilizer N needs of the crop. Table 1 lists in chronological order innovations in proximal sensing techniques based on transmittance and reflectance properties of crop plants as used for guiding fertilizer N management.

### 2.1. Chlorophyll Meters

Transition from remote sensing to proximal sensing was established by Schepers et al. [25] when they used a SPAD meter to measure relative chlorophyll content in the leaves of maize crop grown with a range of fertilizer N levels. As SPAD meter readings very well correlated with rate of applied fertilizer N as well as leaf N concentration, it was suggested that SPAD meter readings can be used to estimate fertilizer N needs of maize crop. Fox et al. [26] found that SPAD meter accurately predicted response of wheat to fertilizer N with lower error rate than that based on N determination in leaf samples. Piekielek and Fox [27] were able to establish a critical SPAD value for maize to distinguish between responsive and non-responsive sites and thus SPAD meters could be used for making decisions on whether to apply fertilizer N or not. It was very useful when manure N was part of the N supply. In developing countries, chlorophyll meters are still being used in this manner even in fields to which very little or no manures is applied [28].

Measured values of the chlorophyll meter are unit less and subject to wide variations due to factors other than N status. To account for this, Schepers et al. [25] proposed creation in each field of a reference strip that was well-fertilized with N because it was found that chlorophyll content and maximum yield plateau similarly with increasing N levels. The SPAD meter reading of the reference strip indicated maximum measured values. Blackmer and Schepers [30] introduced the concept of an N sufficiency index, defined as the ratio of SPAD meter readings for the crops growing in the test field and in a well-fertilized reference strip. Nitrogen sufficiency index values less than 0.95 was used as a criterion to apply additional N fertilizer. Varvel et al. [35] efficiently managed fertilizer N in maize using SPAD meter measurements and following N sufficiency index principles. Chlorophyll meters such as SPAD meter were designed to correlate SPAD readings with leaf chlorophyll content but due to the fact that much of the leaf N is in chlorophyll, these meters became a popular means for predicting the need for additional N in cereals like rice [29,36], maize [27,37,38] and wheat [26,39].

In using chlorophyll meters for estimating leaf N status, there are variations with respect to which leaf to measure and which part of the specific leaf should be used to measure. Argenta et al. [40], Rashid et al. [41], Zhang et al. [42] and Ziadi et al. [43] used chlorophyll meter readings from the topmost leaf in earlier vegetative stages in maize. After the tasseling stage of the crop, Fox et al. [44] and Hawkins et al. [45] used the ear leaf for measurements with SPAD meter. Zhang et al. [46] reported that chlorophyll N content in the first fully expanded maize leaf from the top decreased after silk emergence. But chlorophyll content in the ear leaf increased or did not change. Also, SPAD measurements in maize have been made at one quarter of the distance from the leaf tip toward the stem [44], two thirds [40] or midway between leaf tip and the stalk [42,43]. In rice, the estimated chlorophyll content of the first fully expanded leaf from the top as measured with a SPAD meter served as the best indicator of the N demand [47] and has, therefore, been used to guide the application of a fertilizer N dose at different growth stages of the crop. In South Asia, the center portion of the fully expanded leaf from the top is being used for guiding fertilizer N applications based on SPAD measurements in rice, wheat and maize [28]. In rice, SPAD measurements on the topmost fully expanded leaf to reveal leaf N status have been accepted as a common practice. Nevertheless, reports exist that leaves in lower positions can better separate N level when total N was used as an indicator [48]. SPAD readings of the lower leaves have also been found to be better correlated with the total N in whole leaves and plant [49]. Jinwen et al. [50] observed that SPAD readings of the lower and physiologically older leaves were more sensitive to fertilizer N rates than the upper ones.

### 2.2. Canopy Reflectance Sensors

Canopy reflectance sensors measure visible and NIR spectral reflectance from plant canopies to be interpreted in terms of N stress [51]. Chlorophyll contained in the leaves absorbs between 70% and 90% of all incident light in the red wavelength bands and thus determines visible light reflectance. On the other hand, reflectance of as much as 60% the incident NIR radiation is determined by the structure of mesophyll tissues [52]. Based on early studies showing close correlation between spectral measurements of crop canopies with plant biomass [53,54] and plant N content [55,56,57], it was in mid-1990s that a canopy reflectance sensor was developed by Stone et al. [31]. It measured spectral reflectance from wheat canopy in the red (671 nm) and NIR (780 nm) bands. Based on the data generated by this sensor, the concept of a plant nitrogen spectral index (PNSI) was evolved. The PNSI, the inverse of the NDVI, was found to be highly correlated with estimates of wheat forage N uptake. Using an algorithm (based on the work of Stone et al. [31]) in which N uptake by wheat increased exponentially with PNSI values, Solie et al. [58] used sensor readings to vary fertilizer N rates. This is how technology to variably apply N fertilizer was developed using optical sensing and led to commercial development of the GreenSeeker active sensor in 2001 [24]. The NDVI estimated from reflectance of red and NIR radiation provides information such as photosynthetic efficiency, productivity potential and potential yield [51,59,60,61,62,63,64] and has also been found to be sensitive to leaf area index, green biomass and photosynthetic efficiency [62]. After reviewing a number of algorithms for in-season management of fertilizer N in cereals, Franzen et al. [18] concluded that these are essentially based on active sensors and the concept of NDVI.

Using NDVI measurements of wheat at different times during crop growth period, Raun et al. [32,51] subsequently developed the concepts of response index (estimated as the ratio of NDVI values in the crop relative to those in a well-fertilized reference strip) and potential yield. Raun et al. [32] used potential yield and response index to develop a fertilizer N algorithm for estimating fertilizer N requirement of wheat. The algorithm not only accounted for within-field spatial variability in N supply but also the season-to-season temporal variability in crop performance through the concept of in-season estimated yield (INSEY). Algorithms for estimating potential crop yield and N uptake are now available for many crops and locations around the world [24].

Holland et al. [33] developed an active crop sensor known as Crop Circle, which uses green and NIR bands to estimate N stress and fertilizer N needs of the crop. The NDVI estimated using reflectance of green light is more sensitive to changes in the chlorophyll concentration when crop leaf area index increases beyond 2.0 and potential crop yield than the red NDVI measured by GreenSeeker optical sensor [65,66,67]. This overcame the limitation of using the GreenSeeker sensor at advanced crop growth stages. Both GreenSeeker and Crop Circle sensors were initially developed to be mounted on a tractor to automatically control N application rates on-the-go. Pole mounted versions of these sensors allowed manual measurements of crop reflectance for taking in-season decisions for fertilizer N management. In recent years, GreenSeeker handheld and RapidSCAN CS-45 have been released as handheld versions of these sensors. These are ergonomically designed for ease-of-use, light in mass and are cost effective. These are well suited to manual use, especially in small farms in the developing countries. In these models, the user positions the sensor over plant canopy, pulls the trigger in a slow walking speed and the sensor instantly calculates the vegetation index.

Canopy reflectance sensors cannot directly estimate the amount of N fertilizer needed to overcome crop N stress [68] and need an algorithm to translate sensor readings into fertilizer N requirement. Establishment of reference strips receiving sufficient N fertilizer [32,67,69] allowed development of N response functions as an essential component of the algorithm to translate the sensor readings to the amount of fertilizer N needed by the crop as per expected yield [70]. The reference strips act as local calibration and have to be strategically placed in the fields to account for the spatial variability in yield response to fertilizer N [71].

## 3. Fertilizer N Management in Cereals Using Chlorophyll Meters

Chlorophyll meters estimate relative quantity of chlorophyll. There are at least two types of handheld chlorophyll meters which can be used for assessing crop N status and management of fertilizer N in crops. The most commonly used SPAD 502 Plus chlorophyll meter measures transmittance of light through the leaf at 650 and 940 nm to estimate chlorophyll content (Table 1) [25]. The recently introduced atLEAF chlorophyll meter is similar to SPAD meter but uses a wavelength of 660 nm rather than 650 nm [34]. Readings obtained with an atLEAF chlorophyll meter are very close to those obtained with the SPAD meter but atLEAF chlorophyll meter is currently less expensive than the SPAD meter [34]. However, for N management in cereals, the hand-held Minolta SPAD-502 remains the most used chlorophyll meter and most of the research reported in this review has been carried out by SPAD meter. Yara N-Tester^TM^, a customized version of SPAD-502 chlorophyll meter, is being used to manage fertilizer N in field crops, but mostly in Europe.

Research focused on improving N use efficiency using chlorophyll meters can be divided into two broad groups: (1) Relationship between chlorophyll meter readings and N content of leaves and (2) Establishment and evaluation of the relationship between chlorophyll meter readings and the fertilizer N doses to be top dressed on field crops.

### 3.1. Evaluating Crop N Status Using Chlorophyll Meters

Crop N status can be estimated using a chlorophyll meter but differences in specific leaf weight or leaf thickness can create variations in the relationship between SPAD readings and leaf N content [36,72]. Crop growth stage, cultivars, environmental and stress factors caused by excess or limited water, deficiency of nutrients other than N and pests and diseases constitute other factors which can influence in varying extents the SPAD reading—leaf N status relationship [73].

The relationship between chlorophyll meter readings and leaf N status has generally been found to be linear in maize [25,74,75]. In wheat and rice, it has been found to be non-linear [36,76,77,78,79]. Peng et al. [36] reported improvement in the prediction of leaf N concentration in rice through adjustment of SPAD readings for specific leaf weight. Subsequently, Yang et al. [80], Shukla et al. [78] and Esfahani et al. [79] confirmed the role of adjusting the SPAD readings for specific leaf weights in improving the estimation of plant N accumulation. Peng et al. [47,72], Shukla et al. [78] and Cabangon et al. [81] observed a linear correlation between SPAD values and rice leaf N concentration, measured on a leaf area basis. Esfahani et al. [79] reported that measurements with SPAD meter accounted for about 80% variability in leaf N concentration expressed on basis of leaf area in rice. Similar results were reported by Kyaw [82] in wheat. Parvizi et al. [83] observed highly significant correlations of SPAD readings with leaf N concentrations in wheat. According to Peng et al. [72], to accurately predict plant N status with the SPAD meter, there is a need to develop relationships between SPAD readings and N concentration for different rice cultivars grown under specific climatic conditions. Although Lin et al. [84] reported a positive relationship between SPAD values and leaf N concentration in rice, they also observed that rice variety, growth stage, the leaf position and the measurement point on a leaf blade affected measurements with SPAD meter. Usually a single measurement point in single leaf is used to diagnose plant N status but work of Lin et al. [84] suggests that measurements with SPAD meter should be done at 30 mm to either side of midrib and about one-third of the way down from the leaf tip and averaged.

Mendoza-Tafolla et al. [85] showed that both SPAD and atLeaf chlorophyll meters can estimate the N status of crop plants fairly accurately. A linear relationship between N content of leaves of canola, wheat, barley, potato and corn and measurements with SPAD or atLeaf chlorophyll meters has been reported by Zhu et al. [34]. Recently, Ali et al. [86] reported that atLeaf chlorophyll meter measurements at Feekes 6 stage of wheat leaves explained 55% variation in the N uptake at Feekes 6 stage and 53% variation in grain yield at maturity.

### 3.2. Fertilizer N Management Using Chlorophyll Meters in Different Crops

Chlorophyll meters are very sensitive in the deficient to adequate range of N nutrition. Monitoring chlorophyll content in leaves during the crop growing season can indicate slight N deficiencies early enough to correct them before yield potential is affected. Therefore, managing fertilizer N based on crop need as guided by chlorophyll meter readings can maintain optimal crop N status [47]. There are two ways in which chlorophyll meters can be used to manage fertilizer N in cereal crops. Fixed threshold chlorophyll meter reading or absolute reference value approach involves application of a fertilizer N dose whenever chlorophyll meter reading is found to be less than a threshold reading. Dynamic threshold chlorophyll meter reading approach or sufficiency index approach consists of application of a fertilizer N dose whenever a sufficiency index, defined as chlorophyll meter reading of the plot in question divided by that of a well-fertilized reference plot, falls below a critical value. Despite greater reliability of the sufficiency index approach, the former is more practical in small farms in developing countries as it does not require establishment of a well-fertilized plot.

#### 3.2.1. The Use of Absolute Reference Values

The threshold leaf chlorophyll content expressed in the form of a SPAD threshold reading represents a critical leaf N status below which the crop will suffer from N deficiency and may lead to yield loss [87]. It can be determined from the relationship between SPAD readings and leaf area-based N concentration. The threshold SPAD value is not influenced by excessive N supply and N uptake by crop plants because plant does not produce chlorophyll more than its need regardless of how much N is in the plant [88]. Peng et al. [47] were the pioneers in determining a critical SPAD value for rice that farmers in developing countries in Asia could refer to in the field. A SPAD reading of 35 representing 1.4 g N m^−2^ on leaf area basis was found to be the threshold for guiding N top-dressing in rice variety IR72. Fertilizer N applications were necessary below this threshold value to maintain optimal crop N status and avoid yield loss. The underlying principle in real-time site-specific N management using SPAD meter is that the threshold leaf chlorophyll content or N status must be maintained throughout the cropping season so that a dose of fertilizer N must be applied whenever first fully expanded leaf from the top gives a SPAD reading less than the threshold value. Thus, chlorophyll meter guided N fertilizer applications lead to improved congruence of N supply and crop demand thereby resulting in the production of high grain yields and high fertilizer N use efficiency. An account of research on fertilizer N management in rice and wheat in developing countries based on the absolute chlorophyll meter threshold reading approach is given in the following sub-sections.

##### Rice

On-farm research on using absolute SPAD threshold reference values for fertilizer N management in transplanted wet-seeded rice revealed that for local cultivar groups and climatic conditions in Philippines and Indonesia, the SPAD threshold value of 35 was appropriate in the dry season [87]. But during the wet season with cloudy weather and low radiation and for wet-seeded rice in the dry season, high fertilizer N use efficiency and high yield levels were achieved by applying a dose of fertilizer N whenever SPAD reading of the crop was less the threshold value of 32. In the South and North-west India, threshold SPAD values appropriate for guiding application of fertilizer N to irrigated transplanted rice cultivars were found to be 37 and 37.5, respectively [89,90]. Balasubramanian et al. [91] reported that for distinctly different rice varietal groups, different threshold SPAD readings will have to be used for managing fertilizer N. For example, threshold SPAD reading of 37 or 37.5 worked well for managing fertilizer N in rice cultivars grown in the Indo-Gangetic plain in India [90,92] but the appropriate threshold SPAD reading for the South Indian rice cultivars was 35 [93]. According to Huang et al. [94], optimal SPAD thresholds were 2 units higher in rice varieties with thicker leaves. Hussain et al. [95] found the threshold SPAD reading of 37.5 appropriate for guiding fertilizer N topdressing in the transplanted rice in Pakistan. In Bangladesh, Islam et al. [96] recommended SPAD meter reading of 35 as the threshold value for guiding site-specific N management in transplanted rice. Thus, using 35 as the SPAD threshold, Kyaw [82] obtained significantly higher yields of boro rice with 3–12% less fertilizer N use than by following the general recommendation. Ali et al. [97] reported that fertilizer N management following SPAD threshold of 37 in dry direct-seeded rice produced high yield levels as well as exhibited high fertilizer N use efficiency.

Data compiled in Table 2 show that SPAD meter-based N management in rice leads to significant increases in the N use efficiency when compared with the standard recommendation or the farmers’ fertilizer practices. The increases in the fertilizer use efficiency were recorded generally due to less fertilizer use by following the SPAD meter based strategy as compared to the farmers’ fertilizer practice or the local standard recommendation. Grain yield of rice did not increase by applying fertilizer N doses guided by threshold SPAD meter readings. The SPAD meter based site-specific real-time N management strategy does not lead to the reduction of fertilizer N application at the cost of yield. The fertilizer N management based on the SPAD threshold values not only determines the amount of fertilizer N to be applied but also guides the right time of application of fertilizer N doses. If farmers are using fertilizer N less than the crop requirement, the SPAD meter-based N management will guide application of more fertilizer N with improvement in the yield. As SPAD threshold value guided fertilizer N management in rice ensured application of fertilizer N as per needs of the crop, Chunjiang et al. [98] also observed reduced losses of N to the environment.

The SPAD meter threshold-based fertilizer N management in rice starts two weeks after its transplanting from the nursery to the main field. Balasubramanian et al. [87] and Bijay-Singh et al. [90] observed that there was no need to apply a basal fertilizer N dose if a grain yield of 3 t ha^−1^ or more was obtained in the no-N control plots. It seems obvious because N uptake by rice within 2 weeks of transplanting is very small [99] as seedlings need 7 to 8 days to recover from the transplanting shock [100]. Delaying the first dose of fertilizer N to about two weeks after transplanting of rice or sowing of direct-seeded rice also has the potential to enhance the fertilizer N-use efficiency [78,90,97].

Ali et al. [97] attempted a SPAD meter based fixed-time variable-dose strategy for managing fertilizer N in dry direct-seeded rice. After applying 20 kg N ha^−1^ at 14 days after sowing (DAS) and 30 kg N ha^−1^ at 28 DAS, 30, 40 or 50 kg N ha^−1^ were applied at 49 and 70 DAS depending upon the SPAD meter readings to be ≥40, 40–35 or <35, respectively. The performance of this fixed-time variable-dose strategy was similar to the real-time fertilizer N management strategy based on applying 30 kg N ha^−1^ whenever leaf chlorophyll content was below the SPAD threshold value of 37. Optimum rice yield level along with higher N use efficiency were recorded than when fertilizer N was managed following the general recommendation.

##### Wheat

Unlike in rice, where fertilizer N can be applied at any time up to flowering stage of the crop, N application in wheat is linked with irrigation events. Therefore decisions regarding chlorophyll meter guided fertilizer N applications become complex. Research on fertilizer N management in wheat using SPAD meter is focused on (a) determining the appropriate crop growth stages for mid-season fertilizer N application on the basis of leaf chlorophyll content and (b) developing the criteria based on chlorophyll meter values to apply fertilizer N doses at different crop growth stages coinciding with irrigation events. In wheat, fertilizer N is typically applied in 2 or 3 split doses at sowing and along with first and second irrigation events around 20–25 days after sowing (crown root initiation stage) and 45–50 days after sowing (maximum tillering stage), respectively. Applying a dose of 20 kg N ha^−1^ following a SPAD threshold value of 44 at maximum tillering stage improved the yield of wheat in the lower Gangetic plains in Bangladesh [82]. Hussain et al. [95] found that the SPAD threshold of 42 was appropriate for guiding fertilizer N top dressing in wheat in Pakistan. In the eastern India, where winters are mild and wheat yield levels are relatively lower than those observed in the north-western India and Pakistan, significantly higher fertilizer N use efficiency was observed by applying N as guided by SPAD threshold value of 37 than by managing fertilizer N following the local recommendation [101].

Not only the high yield levels, high protein content in wheat grains also depends upon appropriate fertilizer N management. Takebe et al. [104] observed a close relation between leaf chlorophyll content at the full heading stage and protein content in wheat grains at maturity and recommended the application of 30 kg N ha^−1^ if SPAD meter reading of the leaves at full heading stage was between 50 and 52. In case the SPAD reading at heading stage was between 45 and 50, application 60 kg N ha^−1^ was required to obtain a grain protein content of more than 120 g kg^−1^.

In an acid lateritic soil and under mild winters in the eastern India, Ghosh et al. [105] reported that application of 25 kg N ha^−1^, whenever leaf chlorophyll content was less than the SPAD threshold of 40 up to heading stage, resulted in higher wheat yield levels than those observed by following the local fertilizer N recommendations. Corresponding increases in the agronomic and the recovery efficiencies were 58% and 15%, respectively. SPAD meter cannot be used to guide N topdressing at crown root initiation stage of wheat (about 3 weeks after sowing) because very little variation in leaf color is observed due to application of a basal dose of N at sowing [106]. Also, due to small size of the leaves at this stage, it is difficult to take SPAD meter readings. By conducting a series of experiments, Bijay-Singh [106] concluded that before applying a SPAD meter-guided dose of fertilizer N at the maximum tillering stage, application of 30 kg N ha^−1^ at sowing of wheat and 45 kg N ha^−1^ along with the first irrigation at crown root initiation stage constituted the appropriate prescriptive N management. At the maximum tillering stage, a dose of 30 or 45 kg N ha^−1^ was applied depending upon SPAD reading of the first fully expanded leaf from the top was more than or equal to 42.5 or less than 42.5, respectively. Following this site-specific fertilizer N management strategy, it was possible to produce wheat grain yields at par with those produced with the general recommendation in the region but with higher fertilizer N use efficiency.

Maintenance of optimum leaf chlorophyll content and N status between 50 to 75 days after sowing of wheat is crucial for obtaining high yield levels [107]. Bijay-Singh et al. [90,108] developed a criteria for using SPAD meter readings for guiding tillering stage application of fertilizer N. Based on the data from 3 wheat seasons, statistically significant relationships were observed between grain yield of wheat and the SPAD reading of wheat leaves at the maximum tillering stage [108]. Wheat grain yield response to application of 30 kg N ha^−1^ (over no-N application) at the maximum tillering stage exhibited significant negative linear relationship with SPAD values recorded before applying the fertilizer. Thus, with decreasing SPAD meter readings of the first fully expanded wheat leaves from the top at the maximum tillering stage, wheat grain yield response to applied fertilizer N increased linearly. For example, with SPAD readings of 32.5 and 42.5 at the maximum tillering stage, application of 30 kg N ha^−1^ increased wheat yield by 1.0 or 0.5 t ha^−1^, respectively [108]. Bijay-Singh et al. [90] reported that wheat did not respond to application of 30 kg N ha^−1^ when wheat leaves recorded SPAD meter readings of 44 or more at the maximum tillering stage. However, at SPAD reading of 42, a 20% yield increase was expected by applying a fertilizer N dose of 30 kg ha^−1^.

#### 3.2.2. The Use of Relative Sufficiency Values

When absolute threshold leaf chlorophyll content varies among varietal groups, seasons or regions, dynamic threshold thresholds expressed as percentage of sufficient index is used with SPAD meter-based N management. Hussain et al. [109] monitored sufficiency index in rice at 7- to 10-day interval and whenever SPAD reading of the test plot was less than the threshold 90% of the reading of the N-rich strip, a fertilizer N dose of 30 kg ha^−1^ was applied. Rice yields recorded for different cultivars using the relative sufficiency values were similar to those obtained in the local recommendation treatment but with application of 30 kg ha^−1^ less N. Bijay-Singh [110] also demonstrated the usefulness of this approach for rice cultivars in north-western India. Bijay-Singh et al. [111] followed the 90% sufficiency index approach for guiding fertilizer N application in wet direct-seeded rice in the north-western India and used 50 kg N ha^−1^ less fertilizer than the general recommendation of 120 kg N ha^−1^ in the study area but with no reduction in the grain yield. A significant increase in fertilizer N use efficiency was recorded by managing fertilizer N following dynamic SPAD threshold as per relative sufficiency values [109,110]. Sufficiency index approach has the advantage of being self-calibrating for different soils, seasons and cultivars.

Fertilizer N management following the relative sufficiency approach has also been attempted in wheat. Bijay-Singh [112] applied a basal dose of 30 kg N ha^−1^ at sowing and another 45 kg N ha^−1^ at crown root initiation stage of wheat along with the first irrigation event before applying a fertilizer N dose of 30, 45 or 60 kg ha^−1^ at maximum tillering stage of the crop on the basis of sufficiency index to be more than 90%, between 85 to less than 90% or less than 85%, respectively. Wheat grain yields produced following this strategy were at par with those observed with the general fertilizer N recommendation but with higher fertilizer N use efficiency. In Egypt, Saudy et al. [113] applied fertilizer N to wheat whenever the sufficiency index was less than 95% and recorded higher fertilizer N use efficiency without any yield loss by applying about 40% less fertilizer N than the locally recommended rate of 190 kg N ha^−1^. Yu et al. [114] used 97% sufficiency as the threshold for maize in China.

## 4. Fertilizer Nitrogen Management in Cereals Using Canopy Reflectance Sensors

Estimation of in-season N status in crops is critical for precision N management. Crop N demand and soil N supply are reflected in crop growth status [115]. Thus, fertilizer N application rates are often calculated from N requirement of the crop based on estimates of the expected yield [116]. Active canopy reflectance sensors can help make in-season fertilizer N rate decisions based on expected yield and crop N status. Relationships between sensor measurements and crop N status as well as expected yield of the crop need to be established so that an algorithm can be used to readily interpret sensor measurements in terms of fertilizer N requirement of the crop at the sensed growth stage.

Fairly accurate prediction of N uptake is essential for setting up non-invasive strategies to optimize N fertilization and to reduce the environmental risks associated with injudicious use of fertilizer N. Zhang et al. [117] found that NDVI was exponentially related to leaf N accumulation at Feekes growth stages 4–7 and 8–10 of wheat and explained 68% and 75% of its variability, respectively. Cao et al. [118] conducted studies in the Hebei Province of China and concluded that the three band user configurable Crop Circle ACS-470 sensor can better estimate winter wheat N status as compared to the two fixed band GreenSeeker sensor. At Feekes stage 4–7 of the wheat crop, the GreenSeeker sensor was not able to estimate satisfactorily the plant N concentration but measurements with Crop Circle explained about 60% plant N concentration variability. In Egypt, Ali et al. [86] studied the relation between N uptake and NDVI measured by GreenSeeker optical sensor at Feekes 6 growth stage of wheat and found that GreenSeeker sensor measurements explained the variation in N uptake at Feekes 6 growth stage to the tune of 68.5%. In Brazil, Povh et al. [119] reported a significant correlation between NDVI and foliar N content in wheat, triticale, barley and maize.

GreenSeeker, the most commonly used crop reflectance sensor for precision N management in developing countries, works on an N fertilization optimization algorithm (NFOA) as developed by Raun et al. [32,120] and Mullen et al. [121]. The NFOA follows N-rich strip approach and is based on a ‘mass balance’ calculation of optimum N rate, which actually is the additional N required to increase the potential yield without fertilizer N in a given field to the expected yield estimated from the N-rich strip [122]. Raun et al. [32] gave the concept of potential yield attainable without application of fertilizer N. In the NFOA, it can be estimated from the equations established by relating the in-season estimate of yield (INSEY; estimated by dividing NDVI by growing degree days at the date of sensing) with grain yield from a large number of experimental sites in the study area or using the data from plots with varying N supply established in a small number of experiments. Mullen et al. [121] introduced the concept of in-season response index (RI_NDVI_) defined as the NDVI from N-rich strip divided by the NDVI from the test field. As the RI_NDVI_ was satisfactorily correlated with post-season harvest response index, it allowed estimation of the yield level that can be expected by applying additional N. Thus yield attainable with fertilizer N application is estimated by multiplying the potential yield without fertilizer N with the RI_NDVI_. The NFOA approach is now the commonly reported method for using crop reflectance sensors for fertilizer N management in cereals in developing countries like Mexico [123], China [124], India [125,126,127], Brazil [119] and Egypt [128]. A similar approach was described by Holland and Schepers [129]. It subsequently evolved into one of the manufacturers’ recommended algorithm options for the Crop Circle sensor. Table 3 lists studies conducted in developing countries in which optical sensors have been used for precision fertilizer N management in different crops.

### 4.1. Canopy Reflectance Sensor-Based Fertilizer Nitrogen Management in Rice

In irrigated transplanted rice, fertilizer N is generally applied at three critical growth stages of transplanting, active tillering and panicle initiation. As water on the soil surface interferes in measuring NDVI by canopy reflectance sensor, the sensor-guided fertilizer N dose can be applied only at panicle initiation stage when crop canopy does not allow interference due to water in the field [126,131,136]. In the Indo-Gangetic Plain of South Asia, Ali et al. [137] and Bijay-Singh et al. [126] established robust relationships between INSEY at the panicle initiation stage and yield of rice at maturity. Thus, it became possible to reliably predict the potential yield of the crop using the GreenSeeker sensor. In China, Yao et al. [130] and Xue et al. [131] demonstrated these relationships for rice at tillering and panicle initiation stages. In experiments conducted by Cao et al. [133], rice yield potential and responsiveness to topdressing N application were satisfactorily estimated by the GreenSeeker sensor only at the stem elongation stage. The Crop Circle sensor performed consistently well at stem elongation, booting and heading growth stages.

As GreenSeeker sensor-guided fertilizer N dose can be applied to irrigated transplanted rice only at panicle initiation stage, it is important that appropriate fertilizer N doses are applied at transplanting and active tillering stages of the crop. Bijay-Singh et al. [126] concluded that application of fertilizer N doses of 30 kg ha^−1^ at transplanting and 45 kg ha^−1^ at active tillering stage was the appropriate prescriptive strategy before applying the GreenSeeker-guided N dose at panicle initiation stage. Data from 19 on-farm locations revealed that farmers applied from 92 to 180 kg N ha^−1^ but produced significantly less rice grain yield at 6 out of 19 sites than when fertilizer N was managed using GreenSeeker canopy reflectance sensor. Total fertilizer N application was in the range of 75–97 kg N ha^−1^ under sensor-based N management. Working with dry direct-seeded rice in north-western India, Ali et al. [132] found that panicle initiation stage was the appropriate stage for applying sensor-guided fertilizer N dose. Application of 60 to 90 kg N ha^−1^ in 2 or 3 split doses before applying the sensor-guided N dose produced rice grain yield at par with that produced by following general recommendation. However, total fertilizer N application following sensor-based strategy was less than the general recommendation. Yao et al. [130] conducted three on-farm experiments and revealed that the GreenSeeker sensor-based strategy resulted in a regional optimum N level of 90–110 kg N ha^−1^, of which 45% and 20% N was applied as basal and at tillering stage of the crop. The sensor-guided dose of fertilizer N was applied at stem elongation stage. The partial factor productivity increased by 48% by following the sensor-based N management. Work of Cao et al. [133] revealed that Crop Circle sensor was better suited than GreenSeeker for developing precision N management strategies in high yielding rice cropping systems that require application of as many as four split doses. Xue et al. [131] developed and validated a model based on NDVI measurements with GreenSeeker canopy reflectance sensor, target yield approach and split fertilization strategy to manage fertilizer N in rice. Increased rice yields, lower N rates, higher N use efficiency and higher profits were recorded by using model guided fertilizer management than by following the farmer’s practice.

### 4.2. Canopy Reflectance Sensor-Based Fertilizer Nitrogen Management in Wheat

General fertilizer recommendation for wheat is to apply N at sowing and at crown root initiation stage but farmers have a tendency to apply additional N at the maximum tillering stage which coincides with second irrigation event. At sowing and at crown root initiation stage when crop canopy is very small, it is not possible to measure NDVI using sensors. Therefore, site-specific fertilizer N management for wheat involving canopy reflectance sensors generally consists of applying moderately reduced doses of fertilizer N at sowing and at crown root initiation stage and applying sensor-guided fertilizer N dose at the second or the third irrigation stages. Bijay-Singh et al. [125,127] and Li et al. [124] observed robust relationships between INSEY at the growth stage when fertilizer N is to be applied and the grain yield at maturity. Thus, in-season NDVI measurements with canopy reflectance sensors were used to predict the potential yield without fertilizer N, the expected yield with fertilizer N and the fertilizer N requirement of the crop to achieve the expected yield.

In north-western India, Bijay-Singh et al. [125] reported that before applying a GreenSeeker -guided fertilizer N dose, a total of 90 kg N ha^−1^ was applied in two equal doses at sowing and at crown root initiation stage. Further studies conducted by Bijay-Singh et al. [127] conclusively proved that application of 30 kg N ha^−1^ at sowing and 45 kg N ha^−1^ at crown root initiation stage of wheat constituted a better N management before applying the sensor-guided dose at maximum tillering stage. Wheat grain yields obtained by following sensor-guided N management were at par with those observed with the standard recommendation but with higher recovery efficiency (by 6.7–16.2%) and agronomic efficiency (by 4.7–9.4 kg grain kg N applied^−1^). Sulochna et al. [138] also followed the GreenSeeker based N management in wheat and observed that grain yield was on a par with that observed with the standard recommendation but with higher fertilizer N use efficiency. Thus, applying a moderate amount of fertilizer N at sowing of wheat and enough fertilizer N to meet the high N demand during the period between crown root initiation stage and maximum tillering stage coupled with a sensor-guided fertilizer N dose at maximum tillering stage could ensure not only in high yields but also high N use efficiency. Cao et al. [134] conducted experiments with a winter wheat-summer maize rotation in North China Plain and reported that in both maize and wheat, compared with farmer’s practice and local fertilizer N recommendation, the sensor-guided N management resulted in reduced fertilizer N applications by 62% and 36%, increased N use efficiencies by 68–123% and 20–61%, decreased apparent total N losses by 81% and 57% and lowered intensities of total N_2_O emission, greenhouse gas emission and reactive N losses by 54–68% and 20–42%, respectively. Based on 30 on-farm experiments conducted over two years in North China Plain, Li et al. [124] reported that fertilizer N use efficiencies in winter wheat with fertilizer N management following GreenSeeker canopy reflectance sensor strategy, soil N_min_ approach and farmer practices were 61%, 51% and 13%, respectively.

### 4.3. Canopy Reflectance Sensor Based Fertilizer Nitrogen Management in Maize

Working with rain-fed maize in North-east China, Wang et al. [135] found that rather than GreenSeeker measured NDVI alone, using plant height information together with NDVI improved the prediction of potential yield (with no additional fertilizer N) as well as RI. The improved NFOA (INFOA) based on NDVI combined with relative plant height guided fertilizer N doses for maize better than the NFOA in both black and aeolian sandy soils. The INFOA-based precision N management strategies increased not only marginal returns but also improved N use efficiency and reduced surplus N in the soil. In a calcareous soil in the Nile Delta in Egypt, Ali et al. [127] reported that application of 150 kg N ha^−1^ in two equal split doses at 14 and 30 days after sowing of maize before applying a GreenSeeker canopy reflectance sensor-guided dose produced maize grain yields similar to that obtained by following general recommendation but with less total fertilizer N application.

## 5. Conclusions

More fertilizer N is being used for crop production in the developing than in the developed countries of the world, where majority of the agricultural land is under smallholder farms (less than 2 ha). Smallholders in developing countries have a tendency to achieve high yield levels by using fertilizer N many times even more than the local recommendations, which cannot take into account the dynamic spatial variability in indigenous N supplying capacity of soils. In recent years, handheld versions of chlorophyll meters and canopy reflectance sensors that allow quantitative estimation of leaf N as chlorophyll content through transmission and reflectance of light by the intact leaves through proximal sensing, have become available for use in smallholder farms in developing countries to optimize crop N status. Like variable rate N-fertilizer applicators in large fields, field-specific fertilizer N application in small fields accounts for the variations in the capacity of the soil to supply N.

Measurement of reflectance or transmittance properties of leaves or canopies using proximal sensing can offer great advantages of speed and immediacy in need based fertilizer N application in cereal crops. Whereas chlorophyll meters such as SPAD meter estimate relative chlorophyll content in leaves, the GreenSeeker and the Crop Circle canopy reflectance sensors estimate NDVI from crop canopies. While optical sensors require establishment of N-rich reference strips to relate sensor readings to the fertilizer N requirement, chlorophyll meters can also guide fertilizer N applications without creating N-rich strips.

Chlorophyll meters guide both amount and time of fertilizer N application based on the principle that a threshold chlorophyll or N content must be maintained throughout the crop growth season. It is the most studied and popular mode of using SPAD meter for managing fertilizer N in rice and wheat in smallholder farms in developing countries. By establishing an N-rich strip in the field, chlorophyll meters can also be used following sufficiency index approach for fertilizer N management. It has the advantage of being self-calibrating for different soils, seasons and cultivars.

In-season fertilizer N rate decisions made by active canopy reflectance sensors are based on expected yield and crop N status. Algorithm are now available which can interpret sensor measurements in terms of fertilizer N requirement of the crop at the sensed growth stage. Managing fertilizer N in terms of dose and time of application prior to application of sensor-guided fertilizer N dose is also vital. Studies carried out with rice, wheat and maize in India and China show promising results, but more studies will be needed before canopy reflectance sensors become useful in smallholder farms in developing countries. For using chlorophyll meters and canopy reflectance sensors for fertilizer N management, adequate supply of water and nutrients other than N needs to be maintained because these influence chlorophyll content in the leaves.

Adoption of proximal sensing based N-management strategies using chlorophyll meters and optical sensors will greatly depend upon inclusion of dedicated economic analysis in future investigations [122]. Lack of consistence evidence of economic benefits from these studies will discourage their adoption by smallholder farmers in developing countries. Most of the studies reported so far show that advantage of proximal sensing based N management lies in reduced N rates with almost no increase in yield. Relative benefit from using sensors and chlorophyll meters for fertilizer N management will depend on how good are the current practices [122]. For example, Li et al. [124], who reported a maximum farmers’ rate of over 400 kg N ha^−1^ in winter wheat in North China, could show huge profits in using optical sensor for managing fertilizer N in wheat. Generally cost of technology is also not taken into account. Thus, simple economic analysis for assessing proximal-sensing based N strategies in developing countries, that is based on calculating partial gross margins but does not include additional cost related to the technology, may show average profit increase. It may not be enough to promote fast adoption by farmers.

## Figures and Tables

**Table 1:** Innovations in proximal leaf sensing as used for guiding fertilizer N management in precision agriculture.

Year	Innovation	Reference
1992	SPAD chlorophyll meter (transmission of 650, 940 nm) used to detect N deficiency and estimate fertilizer N needs of maize	Turner and Jund [29], Schepers et al. [25]
1995	Nitrogen sufficiency indices (ratio of SPAD meter readings of the test plot and that of a well-fertilized or N-rich reference plot)	Blackmer and Schepers [30]
1996	Canopy reflectance sensor (reflectance of 671, 780 nm) for detection of variability in plant N stress	Stone et al. [31]
2002	GreenSeeker canopy reflectance sensor (reflectance of 650, 770 nm)	Raun et al. [32], NTech Industries
2004	Crop Circle canopy reflectance sensor (reflectance of 590, 880 nm or 670, 730, 780 nm)	Holland et al. [33], Holland Scientific
2012	atLeaf chlorophyll meter (transmission of 660, 940 nm)	Zhu et al. [34], FT Green LLC

**Table 2:** Effect of soil plant analysis development (SPAD) chlorophyll meter-based fertilizer N management on fertilizer N use efficiency in rice in Asia.

Region	Threshold SPAD Value	Agronomic Efficiency ^†^, kg grain kg N^−1^		Recovery Efficiency ^†^, kg N kg N^−1^		Partial Factor Productivity ^†^, kg grain kg N^−1^		Reference
		Farmer’s Fertilizer Practice ^‡^	SPAD	Farmer’s Fertilizer Practice ^‡^	SPAD	Farmer’s Fertilizer Practice ^‡^	SPAD	
Philippines, Nueva Ecija ^⁋^	35	17.7a ^§^	15.7a	-	-	53.9a	52.2a	Balasubramanian et al. [91]
South India, Old Cauvery Delta	35	18.6a	41.2b	-	-	58.1a	117.2b	
South India, New Cauvery Delta ^#^	35	8.8a	51.0b	-	-	51.6a	118.4b	
Vietnam, Cai Lay District	32	9.8a	17.8b	-	-	33.0a	57.5b	
East India, Nadia, West Bengal	37	24.3a	42.4b	0.43a	0.55b	56.6a	77.3b	Maiti et al. [92] ^‡^
South India, Aduthurai, Old Cauvery Delta	35	13.9a	16.0b	0.39a	0.46b	32.8a	38.0b	Nagarajan et al. [93]
North western India, Punjab	36–37.5	8.8a	16.1b	0.20a	0.30b	34.7a	44.2b	Khurana et al. [102]
North western India, Ludhiana, Punjab	37.5	20.0a	23.7b	0.44a	0.51b	-	-	Bijay-Singh et al. [90] ^‡^
South India, Thanjavur, New Cauvery Delta	35	13.6a	15.0b	0.45a	0.46a	27.9a	31.0b	Nagarajan et al. [93]
South India, Hyderabad, Telangana	37	-	-	-	-	42.8	54.4	Suresh et al. [103]
North western India, Ludhiana, Punjab (dry direct seeded rice)	37	14.1a	22.1b	0.29a	0.35b	40.0a	70.0b	Ali et al. [97]

^†^ Agronomic efficiency of applied N = (grain yield in the N fertilized plot—grain yield in the zero N plot) ÷ total amount of fertilizer N applied; Recovery efficiency of applied N = (total N uptake in grain and straw in the N fertilized plot—total N uptake in grain and straw in the zero N plot) ÷ total amount of fertilizer N applied; Partial factor productivity of applied N = grain yield in the N fertilized plot ÷ total amount of fertilizer N applied. ^‡^ Farmers’ fertilizer practice: All fertilizer management was done by the farmer without any interference by the researcher. However, in some studies conducted on research farms and not in actual farmers’ fields, farmer’s fertilizer practice denotes fixed-schedule N application, for example, in Maiti et al. [92] @100 kg N ha^−1^, in Bijay-Singh et al. [90] @120 kg N ha^−1^ and in Ali et al. [97] @150 kg N ha^−1^. ^§^ For each N use efficiency index (agronomic efficiency, recovery efficiency or partial factor productivity) and site, values with different letters are significantly different by Duncan’s Multiple Range Test at 0.05 probability level (Balasubramanian et al. [91], Maiti et al. [92], Bijay-Singh et al. [90] and Ali et al. [97]), Least Significance Difference at 0.05 probability level (Nagarajan et al. [93]) and *p*-value < 0.001 (Khurana et al. [102]). ^⁋^ Data averaged for 12 farms. ^#^ Data averaged for 20 farms.

**Table 3:** An account of canopy reflectance sensor-based fertilizer N management studies in developing countries.

Site, Crop and Sensor	Salient Findings	Reference
North Plain (China), wheat, GreenSeeker	Averaged across site-years, fertilizer N management following GreenSeeker based strategy, soil Nmin approach and farmer fertilizer practices produced similar grain yields but with total fertilizer N application of 67, 88 and 372 kg ha^−1^, respectively	Li et al. [124]
North western India, wheat, GreenSeeker	At Feekes 5–6 stage of wheat coinciding with 2nd irrigation event, sensor-based estimates of yield and actual wheat yields were strongly correlated. Applying moderate amounts of N at sowing and at crown root initiation stage before applying a sensor-guided dose along with 2nd irrigation produced yield of wheat comparable to that recorded by following local recommendation.	Bijay-Singh et al. [125,127]
Sanjiang Plain (China), rice, GreenSeeker	The sensor-based N management strategy produced rice grain yield similar to that recorded by farmers’ practice but with less total N application	Yao et al. [130]
South China, rice, GreenSeeker	Compared with farmers’ fertilizer practice, the sensor-guided fertilizer N management produced higher yields with reduced N rates and higher N use efficiency.	Xue et al. [131]
North western India, rice, GreenSeeker	Applications of 30 kg N ha^−1^ at transplanting and 45 kg N ha^−1^ at active tillering stages before applying the sensor-guided dose at panicle initiation stage resulted in production of grain yield of rice similar to that observed with general fertilizer recommendation, but with 5.5–21.7% higher N recovery efficiency and 4.7–11.7 kg grain kg N applied^−1^ higher agronomic efficiency	Bijay-Singh et al. [126]
North western India, direct seeded rice, GreenSeeker	Nitrogen recovery efficiency increased by more than 12% by applying sensor-guided fertilizer N dose as compared to when fertilizer N was managed as per standard recommendation	Ali et al. [132]
Sanjiang Plain (China), rice, GreenSeeker and Crop Circle	At the stem elongation stage, both GreenSeeker as well as Crop Circle ACS-470 sensors performed similarly for guiding fertilizer N. However, at booting and heading stages, significantly better fertilizer N recommendation were obtained by the Crop Circle sensor.	Cao et al. [133]
Hebei Province (China), wheat and maize, GreenSeeker	GreenSeeker-based precision N management strategy was consistently better for both wheat and maize in terms of reduced fertilizer N application and higher fertilizer N use efficiency than observed with farmer’s practice and regional optimum N management.	Cao et al. [134]
West Nile Delta (Egypt), maize, GreenSeeker	The appropriate stage for applying a sensor-guided fertilizer N dose in maize was found to be V9. Application of 150 kg N ha^−1^ in two equal splits followed by a sensor-guided dose resulted in increase of 11.6–16.6% recovery efficiency vis-à-vis general recommendation.	Ali et al. [128]
Jilin Province (China), maize, GreenSeeker	In the black and aeolian sandy soils, compared with farmer’s typical N management, the sensor-guided fertilizer N management reduced N surplus by 65% and 62% and improve N use efficiency by 4–40% and 11–65%, respectively.	Wang et al. [135]

## References

[1] Swaminathan M.S. (2007). Can science and technology feed the world in 2025?. Field Crop. Res..

[2] IFADATA http://ifadata.fertilizer.org/ucResult.aspx?temp=20190723054748.

[3] Båge L. (2008). Supporting Smallholders is Crucial to Food Security. As Published in the G8 Summit Special Report of the Financial Times. https://www.ifad.org/web/latest/speech/asset/39036847.

[4] Singh S. (2009). Role of Private and Public Sectors in Supporting Smallholder Rural Enterprises in India: Status, Issues and Alternatives.

[5] (2016). All India Report on Input Survey 2011-12.

[6] Ju X., Gu B., Wu Y., Galloway J.N. (2016). Reducing China’s fertilizer use by increasing farm size. Glob. Environ. Change.

[7] Ren C., Liu S., Van Grinsven H., Reis S., Jin S., Liu H., Gu B. (2019). The impact of farm size on agricultural sustainability. J. Clean. Prod..

[8] Wu Y., Xi X., Tang X., Luo D., Gu B., Lam S.K., Vitousek P.M., Chen D. (2018). Policy distortions, farm size and the overuse of agricultural chemicals in China. Proc. Natl. Acad. Sci. USA.

[9] Wang X., Chen Y., Sui P., Yan P., Yang X., Gao W. (2017). Preliminary analysis on economic and environmental consequences of grain production on different farm sizes in North China Plain. Agric. Syst..

[10] Lesiv M., Laso Bayas J.C., See L., Duerauer M., Dahlia D., Durando N., Hazarika R., Sahariah P.K., Vakolyuk M., Blyshchyk V. (2019). Estimating the global distribution of field size using crowdsourcing. Glob. Chang. Biol..

[11] Ladha J.K., Pathak H., Krupnik T.J., Six J., Van Kessel C. (2005). Efficiency of fertilizer nitrogen in cereal production: Retrospect and prospects. Adv. Agron..

[12] Cassman K.G., Dobermann A., Walters D.T. (2002). Agroecosystems, nitrogen-use efficiency and nitrogen management. AMBIO.

[13] Cassman K.G., Dobermann A., Walters D.T., Yang H. (2003). Meeting cereal demand while protecting natural resources and improving environmental quality. Ann. Rev. Environ. Res..

[14] Bijay-Singh, Singh V.K., Sasaki T. (2017). Advances in nutrient management in rice cultivation. Achieving Sustainable Cultivation of Rice.

[15] Varinderpal-Singh, Bijay-Singh, Yadvinder-Singh, Thind H.S., Gupta R.K. (2010). Need based nitrogen management using the chlorophyll meter and leaf colour chart in rice and wheat in South Asia: A review. Nutr. Cycl. Agroecosyst..

[16] Diacono M., Rubino P., Montemurro F. (2013). Precision nitrogen management of wheat: A review. Agron. Sustain. Dev..

[17] Witt C., Buresh R.J., Peng S., Balasubramanian V., Dobermann A., Fairhurst T.H., Witt C., Buresh R., Dobermann A. (2007). Nutrient management. Rice: A Practical Guide to Nutrient Management.

[18] Franzen D., Kitchen N., Holland K., Schepers J., Raun W. (2016). Algorithms for in-season nutrient management in cereals. Agron. J..

[19] Peng S., Buresh R.J., Huang J., Zhong X., Zou Y., Yang J., Wang G., Liu Y., Hu R., Tang Q. (2010). Improving nitrogen fertilization in rice by site-specific N management. A review. Agron. Sustain. Dev..

[20] Morris T.F., Murrell T., Beegle D.B., Camberato J.J., Ferguson R.B., Grove J., Ketterings Q., Kyveryga P.M., Laboski C.A.M., McGrath J. (2018). Strengths and Limitations of Nitrogen Rate Recommendations for Corn and Opportunities for Improvement. Agron. J..

[21] Fageria N.K., Baligar V.C. (2005). Enhancing nitrogen use efficiency in crop plants. Adv. Agron..

[22] Richardson A.D., Duigan S.P., Berlyn G.P. (2002). An evaluation of non-invasive methods to estimate foliar chlorophyll content. N. Phytol..

[23] Viscarra Rossel R.A., Adamchuk V.I., Sudduth K.A., McKenzie N.J., Lobsey C. (2011). Proximal soil sensing: Updating the pedologist’s toolkit. Adv. Agron..

[24] Mulla D.J. (2013). Twenty five years of remote sensing in precision agriculture: Key advances and remaining knowledge gaps. Biosyst. Eng..

[25] Schepers J.S., Blackmer T.M., Francis D.D., Bock B.R., Kelley K.R. (1992). Predicting N fertilizer needs for corn in humid regions: Using chlorophyll meters. Predicting N Fertilizer Needs for Corn in Humid Regions.

[26] Fox R.H., Piekielek W.P., Macneal K.M. (1994). Using a chlorophyll meter to predict nitrogen fertilizer needs of winter wheat. Commun. Soil Sci. Plant Anal..

[27] Piekielek W.P., Fox R.H. (1992). Use of a chlorophyll meter to predict side-dress nitrogen requirements for maize. Agron. J..

[28] Bijay-Singh, Varinderpal-Singh, Ali A.M. (2020). Site-specific fertilizer nitrogen management in cereals in South Asia. Sustain. Agric. Rev..

[29] Turner F.T., Jund M.F. (1991). Chlorophyll meter to predict nitrogen topdress requirement for semidwarf rice. Agron. J..

[30] Blackmer T.M., Schepers J.S. (1995). Use of a chlorophyll meter to monitor nitrogen status and schedule fertigation for corn. J. Prod. Agric..

[31] Stone M.L., Solie J.B., Raun W.R., Whitney R.W., Taylor S.L., Ringer J.D. (1996). Use of spectral radiance for correcting in-season fertilizer nitrogen deficiencies in winter wheat. Trans. ASAE.

[32] Raun W.R., Solie J.B., Johnson G.V., Stone M.L., Mullen R.W., Freeman K.W., Thomason W., Lukina E.V. (2002). Improving nitrogen use efficiency in cereal grain production with optical sensing and variable rate application. Agron. J..

[33] Holland K.H., Schepers J.S., Shanahan J.F., Horst G.L. Plant canopy sensor with modulated polychromatic light. Proceedings of the 7th International Conference on Precision Agriculture.

[34] Zhu J., Tremblay N., Liang Y. (2012). Comparing SPAD and atLEAF values for chlorophyll assessment in crop species. Cand. J. Soil Sci..

[35] Varvel G.E., Schepers J.S., Francis D.D. (1997). Ability for in-season correction of nitrogen deficiency in corn using chlorophyll meters. Soil Sci. Soc. Am. J..

[36] Peng S., Garcia F.V., Laza R.C., Cassman K.G. (1993). Adjustment for specific leaf weight improves chlorophyll meter’s estimate of rice leaf nitrogen concentration. Agron. J..

[37] Blackmer T.M., Schepers J.S. (1994). Techniques for monitoring crop nitrogen status in corn. Commun. Soil Sci. Plant Anal..

[38] Piekielek W.P., Fox R.H., Toth J.D., Macneal K.E. (1995). Use of a chlorophyll meter at the early dent stage of corn to evaluate N sufficiency. Agron. J..

[39] Follet R.H., Follet R.F., Halvorson A.D. (1992). Use of a chlorophyll meter to evaluate the nitrogen status of dryland winter wheat. Commun. Soil Sci. Plant Anal..

[40] Argenta G., Silva P., Sangoi L. (2004). Leaf relative chlorophyll content as an indicator parameter to predict nitrogen fertilization in maize. Ciência Rural.

[41] Rashid M.T., Voroney P., Parkin G. (2005). Predicting nitrogen fertilizer requirements for corn by chlorophyll meter under different N availability conditions. Can J. Soil Sci..

[42] Zhang J., Blackmer A.M., Blackmer T.M. (2008). Differences in physiological age affect diagnosis of nitrogen deficiencies in cornfields. Pedosphere.

[43] Ziadi N., Brassard M., Belanger G., Claessens A., Tremblay N., Cambouris A.N., Nolin M.C., Parent L. (2008). Chlorophyll measurements and nitrogen nutrition index for the evaluation of corn nitrogen status. Agron. J..

[44] Fox R.H., Piekielek W.P., Macneal K.E. (2001). Comparison of late-season diagnostic tests for predicting nitrogen status of corn. Agron. J..

[45] Hawkins J.A., Sawyer J.E., Barker D.W., Lundvall J.P. (2007). Using relative chlorophyll meter values to determine nitrogen application rates for corn. Agron. J..

[46] Zhang J., Blackmer A.M., Blackmer T.M. (2009). Reliability of chlorophyll meter measurements prior to corn silking as affected by the leaf change problem. Commun. Soil Sci. Plant Anal..

[47] Peng S., Garcia F.V., Laza R.C., Sanico A.L., Visperas R.M., Cassman K.G. (1996). Increased N-use efficiency using a chlorophyll meter on high-yielding irrigated rice. Field Crop. Res..

[48] Zhou Q.F., Wang J.H. (2003). Comparison of upper leaf and lower leaf of rice plants in response to supplemental nitrogen levels. J. Plant Nutr..

[49] Li G.H., Xue L.H., You J., Wang S.H., Ding Y.F., Wu H., Yang W.X. (2007). Spatial distribution of leaf N content and SPAD value and determination of the suitable leaf for N diagnosis in rice. Agric. Sci. China.

[50] Li J., Yang J., Fei P., Song J., Li D., Ge C., Chen W. (2009). Responses of rice leaf thickness, SPAD readings and chlorophyll a/b ratios to different nitrogen supply rates in paddy field. Field Crop. Res..

[51] Raun W.R., Solie J.B., Johnson G.V., Stone M.L., Lukina E.V., Thomason W.E., Schepers J.S. (2001). In-season prediction on potential grain yield in winter wheat using canopy reflectance. Agron. J..

[52] Campbell J.B. (2002). Introduction to Remote Sensing.

[53] Kleman J., Fagerlund E. (1987). Influence of different nitrogen and irrigation treatments on the spectral reflectance of barley. Remote Sens. Environ..

[54] Wanjura D.F., Hatfield J.L. (1987). Sensitivity of spectral vegetative indices to crop biomass. Trans. ASAE.

[55] Walburg G.M.M.E., Bauer M.E., Daughtry C.S.T., Housley T.L. (1982). Effects of nitrogen nutrition on the growth, yield and reflectance characteristics of corn canopies. Agron. J..

[56] Peñuelas J., Gamon J.A., Fredeen A.L., Merino J., Field C.B. (1994). Reflectance indices associated with physiological changes in nitrogen- and water-limited sunflower leaves. Remote Sens. Environ..

[57] Ma B.L., Morrison M.J., Dwyer L. (1996). Canopy light reflectance and field greenness to assess nitrogen fertilization and yield of maize. Agron. J..

[58] Solie J.B., Raun W.R., Whitney R.W., Stone M.L., Ringer J.D. (1996). Optical sensor based field element size and sensing strategy for nitrogen application. Trans. ASAE.

[59] Thenkabail P.S., Smith R.B., DePauw E. (2000). Hyperspectral vegetation indices and their relationships with agricultural crop characteristics. Remote Sens. Environ..

[60] Ma B.L., Dwyer L.M., Costa C., Cober E.R., Morrison M.J. (2001). Early prediction of soybean yield from canopy reflectance measurements. Agron. J..

[61] Báez-González A.D., Chen P., Tiscareño-López M., Srinivasan R. (2002). Using satellite and field data with crop growth modeling to monitor and estimate maize yield in Mexico. Crop Sci..

[62] Aparicio N., Villegas D., Araus J.L., Casadesús J., Royo C. (2002). Relationship between growth traits and spectral vegetation indices in durum wheat. Crop Sci..

[63] Teal R.K., Tubana B., Girma K., Freeman K.W., Arnall D.B., Walsh O., Raun W.R. (2006). In-season prediction of corn grain yield potential using normalized difference vegetation index. Agron. J..

[64] Fox R.H., Walthall C.L., Schepers J.S., Raun W.R. (2008). Crop monitoring technologies to assess nitrogen status. Nitrogen in Agricultural Systems.

[65] Shanahan J., Schepers J.S., Francis D.D., Varvel G.E., Wilhelm W.W., Tringe J.M., Schlemmer M.R., Major D.J. (2001). Use of Remote-Sensing Imagery to Estimate Corn Grain Yield. Agron. J..

[66] Sripada R.P., Heiniger R.W., White J.G., Weisz R. (2006). Aerial color infrared photography for determining late-season nitrogen requirements in corn. Agron. J..

[67] Sripada R.P., Schmidt J.P., Dellinger A.E., Beegle D.B. (2008). Evaluating multiple indices from a canopy reflectance sensor to estimate corn N requirements. Agron. J..

[68] Samborski S.M., Tremblay N., Fallon E. (2009). Strategies to make use of plant sensors-based diagnostic information for nitrogen recommendations. Agron. J..

[69] Kitchen N.R., Sudduth K.A., Drummond S.T., Scharf P.C., Palm H.L., Roberts D.F., Vories E.D. (2010). Ground-Based Canopy Reflectance Sensing for Variable-Rate Nitrogen Corn Fertilization. Agron. J..

[70] Scharf P.C., Shannon D.K., Palm H.L., Sudduth K.A., Drummond S.T., Kitchen N.R., Mueller L.J., Hubbard V.C., Oliveira L.F. (2011). Sensor-Based Nitrogen Applications Out-Performed Producer-Chosen Rates for Corn in On-Farm Demonstrations. Agron. J..

[71] Mamo M., Malzer G.L., Mulla D.J., Huggins D.J., Strock J. (2003). Spatial and temporal variation in economically optimum N rate for corn. Agron. J..

[72] Peng S., Laza R.C., Garcia F.C., Cassman K.G. (1995). Chlorophyll meter estimates leaf area-based N concentration of rice. Commun. Soil Sci. Plant Anal..

[73] Smeal D., Zhang H. (1994). Chlorophyll meter evaluation for nitrogen management in corn. Commun. Soil Sci. Plant Anal..

[74] Chapman S.C., Barreto H.J. (1997). Using a chlorophyll meter to estimate specific leaf nitrogen of tropical maize during vegetative growth. Agron. J..

[75] Bullock D.G., Anderson D.S. (1998). Evaluation of the Minolta SPAD-502 chlorophyll meter for nitrogen management in corn. J. Plant Nutr..

[76] Chubachi T., Asano I., Oikawa T. (1986). The diagnosis of nitrogen nutrition of rice plant (Sasanishki) using chlorophyll meter. Jpn. J. Soil Sci. Plant Nutr..

[77] Debaeke P., Rouet P., Justes E. (2006). Relationship between the normalized SPAD index and the NNI: Application to durum wheat. J. Plant Nutr..

[78] Shukla A.K., Ladha J.K., Singh V.K., Dwivedi B.S., Balasubramanian V., Gupta R.K., Sharma S.K., Singh Y., Pathak H., Pandey P.S. (2004). Calibrating the leaf color chart for nitrogen management in different genotypes of rice and wheat in a systems perspective. Agron. J..

[79] Esfahani M., Abbasi H.A., Rabiei B., Kavousi M. (2008). Improvement of nitrogen management in rice paddy fields using chlorophyll meter (SPAD). Paddy Water Environ..

[80] Yang W.H., Peng S., Huang J., Sanico A.L., Buresh R.J., Witt C. (2003). Using leaf colour charts to estimate leaf nitrogen status of rice. Agron. J..

[81] Cabangon R.J., Castillo E.G., Tuong T.P. (2011). Chlorophyll meter-based nitrogen management of rice grown under alternate wetting and drying irrigation. Field Crop. Res..

[82] Kyaw K.K., Vlek P.L.G., Denich M., Martius C., Giesen N.V.D. (2003). Plot-Specific N Fertilizer Management for Improved N-Use Efficiency in Rice Based Systems of Bangladesh.

[83] Parvizi Y., Ronaghi A., Maftoun M., Karimian N. (2004). Growth, nutrient status and chlorophyll meter readings in wheat as affected by nitrogen and manganese. Commun. Soil Sci. Plant Anal..

[84] Lin F.F., Deng J.S., Shi Y.Y., Chen L.S., Wang K. (2010). Investigation of SPAD meter-based indices for estimating rice nitrogen status. Comput. Electron. Agric..

[85] Mendoza-Tafolla R.O., Juarez-Lopez P., Ontiveros-Capurata R.E., Sandoval-Villa M., Alia-Tejacal I., Alejo-Santiago G. (2019). Estimating Nitrogen and Chlorophyll Status of Romaine Lettuce Using SPAD and at LEAF Readings. Not. Bot. Horti Agrobot. Cluj Napoca.

[86] Ali A.M., Ibrahim S.M., Bijay-Singh (2019). Wheat grain yield and nitrogen uptake prediction using atLeaf and GreenSeeker portable optical sensors at jointing growth stage. Inf. Process. Agric..

[87] Balasubramanian V., Morales A.C., Cruz R.T., Abdulrachman S. (1999). On-farm adaptation of knowledge-intensive nitrogen management technologies for rice systems. Nutr. Cycl. Agroecosyst..

[88] Peterson T.A., Blackmer T.M., Francis D.D., Schepers J.S. (1993). Using a Chlorophyll Meter to Improve N Management.

[89] IRRI-CREMNET (Crop and Resource Management Network) (1998). Progress Report for 1997.

[90] Bijay-Singh, Yadvinder-Singh, Ladha J.K., Bronson K.F., Balasubramanian V., Singh J., Khind C.S. (2002). Chlorophyll meter and leaf color chart-based nitrogen management for rice and wheat in northwestern India. Agron. J..

[91] Balasubramanian V., Morales A.C., Cruz R.T., Thiyagarajan T.M., Nagarajan R., Babu M. (2000). Adaptation of the chlorophyll meter (SPAD) technology for real-time N management in rice: A review. Int. Rice Res. Notes.

[92] Maiti D., Das D.K., Karak T., Banerjee M. (2004). Management of nitrogen through the use of leaf color chart (LCC) and soil plant analysis development (SPAD) or chlorophyll meter in rice under irrigated ecosystem. Sci. World J..

[93] Nagarajan R., Ramanathan S., Muthukrishnan P., Stalin P., Ravi V., Babu M., Selvam S., Sivanatham M., Dobermann A., Witt C., Doberman A., Witt C., Dawe D. (2004). Site-specific nutrient management in irrigated rice systems of Tamil Nadu, India. Increasing Productivity of Intensive Rice Systems through Site-Specific Nutrient Management.

[94] Huang J., He F., Cui K., Buresh R.J., Xu B., Gong W., Peng S. (2008). Determination of optimal nitrogen rate for rice varieties using a chlorophyll meter. Field Crop. Res..

[95] Hussain F., Zia M.S., Akhtar M.E., Yasin M. (2003). Nitrogen management and use efficiency with chlorophyll meter and leaf colour chart. Pak. J. Soil Sci..

[96] Islam M.S., Bhuiya M.S., Rahman S., Hussain M.M. (2009). Evaluation of SPAD and LCC based nitrogen management in rice (*Oryza sativa* L.). Bangladesh J. Agric. Res..

[97] Ali A.M., Thind H.S., Sharma S., Yadvinder-Singh (2015). Site-specific nitrogen management in dry direct-seeded rice using chlorophyll meter and leaf colour chart. Pedosphere.

[98] Zhao C., Jiang A., Huang W., Liu K., Liu L., Wang J. (2007). Evaluation of variable-rate nitrogen recommendation of winter wheat based on SPAD chlorophyll meter measurement. N. Z. J. Agric. Res..

[99] Peng S., Cassman K.G. (1998). Upper threshold of nitrogen uptake rates and associated nitrogen fertilizer efficiencies in irrigated rice. Agron. J..

[100] Meelu O.P., Gupta R.K. (1980). Time of fertilizer nitrogen application in rice culture. Int. Rice Res. Newsl..

[101] Maiti D., Das D.K. (2006). Management of nitrogen through the use of Leaf Colour Chart (LCC) and Soil Plant Analysis Development (SPAD) in wheat under irrigated ecosystem. Arch. Agron. Soil Sci..

[102] Khurana H.S., Phillips S.B., Bijay-Singh, Dobermann A., Sidhu A.S., Yadvinder-Singh, Peng S. (2007). Performance of site-specific nutrient management for irrigated, transplanted rice in Northwest India. Agron. J..

[103] Suresh M., Balaguravaiah D., Jayasree G. (2017). Calibrating the leaf colour chart and SPAD based nitrogen management on leaf N content and yield in rice. Int. J. Pure Appl. Biosci..

[104] Takebe M., Okazaki K., Karasawa T., Watanabe J., Ohshita Y., Tsuji H. (2006). Leaf colour diagnosis and nitrogen management for winter wheat ‘‘Kitanokaori’’ in Hokkaido. Japan. J. Soil Sci. Plant Nutr..

[105] Ghosh M., Swain D.K., Jha M.K., Tewari V.K. (2018). Chlorophyll meter-based nitrogen management of wheat in eastern India. Exp. Agric..

[106] Bijay-Singh, Varinderpal-Singh, Yadvinder-Singh, Kumar A., Sharma S., Thind H.S., Choudhary O.P., Vashistha M. (2018). Site-specific fertilizer nitrogen management in irrigated wheat using chlorophyll meter (SPAD meter) in the north-western India. J. Indian Soc. Soil Sci..

[107] Islam M.R., Haque K.S., Akter N., Karim M.A. (2014). Leaf chlorophyll dynamics in wheat based on SPAD meter reading and its relationship with grain yield. Sci. Agric..

[108] Bijay-Singh, Varinderpal-Singh, Yadvinder-Singh, Thind H.S., Kumar A., Satinderpal-Singh, Choudhary O.P., Gupta R.K., Vashistha M. (2013). Supplementing fertilizer nitrogen application to irrigated wheat at maximum tillering stage using chlorophyll meter and optical sensor. Agric. Res..

[109] Hussain F., Bronson K.F., Yadvinder-Singh, Bijay-Singh, Peng S. (2000). Use of chlorophyll meter sufficiency indices for nitrogen management of irrigated rice in Asia. Agron. J..

[110] Bijay-Singh (2008). Crop demand-driven site specific nitrogen applications in rice (*Oryza sativa*) and wheat (*Triticum aestivum*): Some recent advances. Indian J. Agron..

[111] Bijay-Singh, Gupta R.K., Yadvinder-Singh, Gupta S.K., Jagmohan-Singh, Bains J.S., Vashistha M. (2006). Need-based nitrogen management using leaf color chart in wet direct seeded rice in North Western India. J. N. Seeds.

[112] Bijay-Singh (2012). Plant-Need Based Nitrogen Management in Rice and Wheat.

[113] Saudy H.S. (2014). Chlorophyll meter as a tool for forecasting wheat nitrogen requirements after application of herbicides. Arch. Agron. Soil Sci..

[114] Yu W., Miao Y., Feng G., Yue S., Liu B. Evaluating different methods of using chlorophyll meter for diagnosing nitrogen status of summer maize. Proceedings of the First International Conference on Agro-Geoinformatics.

[115] Schröder J.J., Neeteson J.J., Enema O., Struik P.C. (2000). Does the crop or the soil indicate how to save nitrogen in maize production? Reviewing the state of the art. Field Crop. Res..

[116] Lemaire G., Jeuffroy M.H., Gastal F. (2008). Diagnosis tool for plant and crop N status in vegetative stage: Theory and practices for crop N management. Eur. J. Agron..

[117] Zhang J., Liu X., Liang Y., Cao Q., Tian Y., Zhu Y., Cao W., Liu X. (2019). Using a Portable Active Sensor to Monitor Growth Parameters and Predict Grain Yield of Winter Wheat. Sensors.

[118] Cao Q., Miao Y., Feng G., Gao X., Li F., Liu B., Yue S., Cheng S., Ustin S.L., Khosla R. (2015). Active canopy sensing of winter wheat nitrogen status: An evaluation of two sensor systems. Comput. Electron. Agric..

[119] Povh F.P., Molin J.P., Gimenez L.M., Pauletti V., Molin R., Salvi J.V. (2008). Comportamento do NDVI obtido por sensor ótico ativo em cereais. Pesquisa Agropecuária Brasileira.

[120] Raun W.R., Solie J.B., Stone M.L., Martin K.L., Freeman K.W., Mullen R.W., Zhang H., Schepers J.S., Johnson G.V. (2005). Optical sensor-based algorithm for crop nitrogen fertilization. Commun. Soil Sci. Plant Anal..

[121] Mullen R.W., Freeman K.W., Raun W.R., Johnson G.V., Stone M.L., Solie J.B. (2003). Identifying an in-season response index and the potential to increase wheat yield with nitrogen. Agron. J..

[122] Colaço A.F., Bramley R.G.V. (2018). Do crop sensors promote improved nitrogen management in grain crops?. Field Crop. Res..

[123] Ortiz-Monasterio J.I., Raun W.R. (2007). Reduced nitrogen and improved farm income for irrigated spring wheat in the Yaqui Valley, Mexico, using sensor based nitrogen management. J. Agric. Sci. Camb..

[124] Li F., Miao Y., Zhang F., Cui Z., Li R., Chen X., Zhang H., Schroder J., Raun W.R., Jia L. (2009). In-season optical sensing improves nitrogen-use efficiency for winter wheat. Soil Sci. Soc. Am. J..

[125] Bijay-Singh, Sharma R.K., Kaur J., Jat M.L., Martin K.L., Yadvinder-Singh, Varinderpal-Singh, Chandna P., Choudhary O.P., Gupta R.K. (2011). Assessment of the nitrogen management strategy using an optical sensor for irrigated wheat. Agron. Sustain. Dev..

[126] Bijay-Singh, Varinderpal-Singh, Purba J., Sharma R.K., Jat M.L., Yadvinder-Singh, Thind H.S., Gupta R.K., Choudhary O.P., Chandna P. (2015). Site-specific nitrogen management in irrigated transplanted rice (*Oryza sativa*) using an optical sensor. Precis. Agric..

[127] Bijay-Singh, Varinderpal-Singh, Yadvinder-Singh, Thind H.S., Kumar A., Choudhary O.P., Gupta R.K., Vashistha M. (2017). Site-specific fertilizer nitrogen management using optical sensor in irrigated wheat in the north-western India. Agric. Res..

[128] Ali A.M., Abou-Amer I., Ibrahim S.M. (2018). Using GreenSeeker active optical sensor for optimizing maize nitrogen fertilization in calcareous soils of Egypt. Arch. Agron. Soil Sci..

[129] Holland K.H., Schepers J.S. (2010). Derivation of a variable rate nitrogen application model for in-season fertilization of corn. Agron. J..

[130] Yao Y., Miao Y., Huang S., Gao L., Ma X., Zhao G., Jiang R., Chen X., Zhang F., Yu K. (2012). Active canopy sensor-based precision N management strategy for rice. Agron. Sustain. Dev..

[131] Xue L., Li G., Qin X., Yang L., Zhang H. (2014). Topdressing nitrogen recommendation for early rice with an active sensor in South China. Precis. Agric..

[132] Ali A.M., Thind H.S., Varinderpal-Singh, Bijay-Singh (2015). A framework for refining nitrogen management in dry direct-seeded rice using GreenSeeker™ optical sensor. Comput. Electron. Agric..

[133] Cao Q., Miao Y., Shen J., Yu W., Yuan F., Cheng S., Huang S., Wang H., Yang W., Liu F. (2016). Improving in-season estimation of rice yield potential and responsiveness to topdressing nitrogen application with Crop Circle active crop canopy sensor. Precis. Agric..

[134] Cao Q., Miao Y., Feng G., Gao X., Liu B., Liu Y., Li F., Khosla R., Mulla D.J., Zhang F. (2017). Improving nitrogen use efficiency with minimal environmental risks using an active canopy sensor in a wheat-maize cropping system. Field Crop. Res..

[135] Wang X., Miao Y., Dong R., Chen Z., Guan Y., Yue X., Fang Z., Mulla D.J. (2019). Developing Active Canopy Sensor-Based Precision Nitrogen Management Strategies for Maize in Northeast China. Sustainability.

[136] Nguyen H.T., Lee K., Lee B.W. (2008). Recommendation of nitrogen topdressing rates at panicle initiation stage of rice using canopy reflectance. J. Crop Sci. Biotech..

[137] Ali A.M., Thind H.S., Sharma S., Varinderpal-Singh (2014). Prediction of dry direct-seeded rice yields using chlorophyll meter, leaf color chart and GreenSeeker optical sensor in northwestern India. Field Crop. Res..

[138] Alam M.P., Ali N., Singh S.K. (2018). Precision nitrogen management on nutrient uptake and nitrogen use efficiency in irrigated wheat. Curr. J. App. Sci. Technol..

